# Ozone Exposure Controls Oxidative Stress and the Inflammatory Process of Hepatocytes in Murine Models

**DOI:** 10.3390/antiox13020212

**Published:** 2024-02-08

**Authors:** Silvania Mol Pelinsari, Mariáurea Matias Sarandy, Emerson Ferreira Vilela, Rômulo Dias Novaes, Jade Schlamb, Reggiani Vilela Gonçalves

**Affiliations:** 1 Departament of General Biology, Federal University of Viçosa, Viçosa 36570-900, MG, Brazil; silvania.pelinsari@ufv.br (S.M.P.);; 2 Plants for Human Health Institute, North Carolina Research Campus, North Carolina State University, Kannapolis, NC 28081, USA; 3 Agriculture and Livestock Research Enterprise of Minas Gerais (EPAMIG-Sudeste), Viçosa 36570-000, MG, Brazil; 4 Departament of Structural Biology, Federal University of Alfenas, Alfenas 37130-001, MG, Brazil; romulo.novaes@unifal-mg.edu.br; 5 Departament of Animal Biology, Federal University of Viçosa, Viçosa 36570-900, MG, Brazil

**Keywords:** ozone exposure, oxidative stress, antioxidant enzymes, liver, inflammation

## Abstract

(1) Background: Ozone exposure is a promising tool for treating liver damage since it is known to control the release of free radicals and increase the expression of antioxidant enzymes. The objective is to investigate the main intracellular pathways activated after exposure to ozone, considering the dosage of antioxidant enzymes and markers of oxidative stress. (2) Methods: This systematic review was performed based on the PRISMA guidelines and using a structured search in MEDLINE (PubMed), Scopus, and Web of Science. Bias analysis and methodological quality assessments were examined using the SYRCLE Risk of Bias tool. (3) Results: Nineteen studies were selected. The results showed that the exposure to ozone has a protective effect on liver tissue, promoting a decrease in inflammatory markers and a reduction in oxidative stress in liver tissue. In addition, ozone exposure also promoted an increase in antioxidant enzymes. The morphological consequences of controlling these intracellular pathways were reducing the tissue inflammatory process and reducing areas of degeneration and necrosis. (4) Conclusions: Ozone exposure has a beneficial effect on models of liver injury through the decrease in oxidative stress in tissue and inflammatory markers. In addition, it regulates the Nrf2/ARE antioxidant pathway and blocks the NF-κB inflammatory pathway.

## 1. Introduction

Liver diseases are responsible for 2 million deaths per year worldwide [1]. Generally, chronic liver diseases such as non-alcoholic fatty liver disease (NAFLD) and chronic viral hepatitis B and C are associated with inflammation and oxidative stress in the tissue [2,3]. Oxidative stress affects major cellular components and is commonly emphasized in the pathogenesis of various degenerative and chronic diseases, which can result in serious damage to the human body [3]. When the excessive production of free radicals and reactive oxygen species (ROS) occurs, changes can be made in proteins, lipids and DNA cells. Consequently, it promotes degeneration and cell death (apoptosis or necrosis).

Due to its metabolic activity, the liver constitutes an organ that is particularly susceptible to oxidative stress. When this tissue undergoes damage, it can result in an imbalance of redox metabolism and the activation of important inflammatory pathways like early growth response factor 1 (Egr-1), nuclear factor-kappa B (NF-κB), and activator protein-1 (AP-1) [4]. The activation of these pathways promotes the expression of inflammatory markers that attract more defense cells, like macrophages and neutrophils, creating a pro-oxidant feedback environment. Within hepatocyte cell damage, the electrons inside the mitochondria are transported, slowly increasing the chance of generating ROS [2]. This pathological chain reaction exposes the liver to great oxidative stress and can result in the death of hepatocytes by necrosis or apoptosis [4]. However, the mechanisms involved in this damage process remain poorly understood, and more investigations are necessary to understand the real relationship between the main pathways activated and the interrelation among them.

Considering the crucial role of oxidative stress and subsequently the inflammatory process in liver diseases, antioxidant therapies are considered a great option for treating liver disorders. In this context, the search for alternative therapies that reduce liver damage has grown substantially [5]. Therefore, ozone exposure has become a promising tool because it works by stimulating the immune system locally and systemically, leading to a fast and safe tissue repair process [5]. Ozone exposure uses a gas mixture with 5% ozone (O_3_) and 95% oxygen (O_2_). The ozone (O_3_) is a molecule composed of three oxygen atoms, including a stable pair (O_2_) and an unstable third atom, which gives ozone its beneficial effects [6]. Ozone has a broad range of actions, encompassing immunoregulatory and anti-inflammatory properties, antioxidant activity, antimicrobial effects, contribution to analgesia and vasodilation, and the promotion of blood flow and oxygenation, as well as serving as a modulator in regenerative processes and epigenetic modifications [7]. Ozone treatment is non-invasive, non-pharmacological, and devoid of side effects, based on the regenerative capacity of ozone applied in medicine. It is used in over 50 pathological processes, including cardiovascular, peripheral vascular, neurological, degenerative, orthopedic, gastrointestinal, and genitourinary conditions, as well as in multiple sclerosis, fibromyalgia, skin diseases/wound healing, infectious diseases, and dentistry [7]. However, in a study, contrary to expectations, ozone was observed to negatively affect the hepatic damage induced by iron, showing a synergistic effect as it increased periportal inflammation [5].

Several studies have indicated that ozone exposure promotes the activation of the nuclear factor erythroid 2-related factor 2 (Nrf2) pathway. This pathway increases antioxidant enzyme expression, reduces pro-inflammatory cytokine levels [8,9], and increases cellular adaptation to oxidative stress [8]. Nrf2 represents a crucial regulator of cellular defense, controlling the expression of antioxidant genes that ultimately exert anti-inflammatory functions. Nrf2 is proven to contribute to the regulation of the heme oxygenase-1 (HO-1) axis, NF-κB pathway, and macrophage metabolism [10].

Therefore, the oxidative preconditioning generated in liver cells by ozone exerts a protective effect by stimulating the endogenous antioxidant system, and consequently by the stimulus, the anti-inflammatory system [8]. However, the study’s outcomes remain inconclusive and controversial, reinforcing the importance of performing a critical analysis of available evidence. Considering that current evidence is based on fragmented data, a better understanding of the pathways and mechanisms cellularly activated after ozone exposure on oxidative stress in liver tissue is essential. Thus, we used a systematic review framework to integrate the pre-clinical evidence (in vivo) to investigate the relevance of ozone exposure in the treatment of liver diseases with a focus on oxidative balance mechanisms and their relationship with the inflammatory process. We believe mapping signaling pathways may contribute to broadening the understanding of the mechanisms involved in ozone exposure that relate to oxidative stress and inflammation in liver tissue damage. The methodological quality of the studies was reviewed, and the risk of bias associated with the current evidence was also critically analyzed.

## 2. Materials and Methods

### 2.1. Focus Question

The main question to be answered in this systematic review is as follows: what is the influence of ozone exposure on oxidative stress in liver tissue in murine models?

### 2.2. Search Strategy

**Figure 1:**
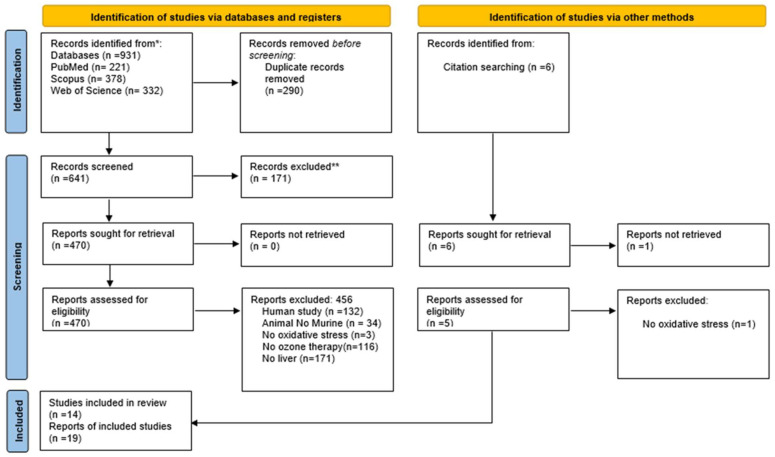
PRISMA diagram—* Consider, if feasible to do so, reporting the number of records identified from each database or register searched (rather than the total number across all databases/registers). ** If automation tools were used, indicate how many records were excluded by a human and how many were excluded by automation tools [11]. For more information, visit http://www.prisma-statement.org (accessed on 7 April 2021). Different phases of the selection of studies for conducting qualitative and quantitative analyses. Flow diagram of the systematic review literature search results. Based on “Preferred Reporting Items for Systematic Reviews and Meta-Analyses: The PRISMA Statement”. http://www.prisma-statement.org.

This systematic review was conducted based on the PRISMA guidelines (preferred reporting items for systematic reviews and meta-analysis) (Figure 1), which were used as a guide for study selection, screening, and eligibility [11]. The protocol details for this systematic review were registered in the Prospective International Registry of Systematic Reviews (PROSPERO: CRD42021264362). Details of the Population, Intervention, Comparators, and Outcomes (PICO) can be found in Table S1. An extensive literature search was carried out using the electronic databases Medline/PubMed (https://www.ncbi.nlm.nih.gov/pubmed), Scopus (https://www.scopus.com/home.URI), and Web of Science (https://www-periodicos-capes-gov-br.ezl.periodicos.capes.gov.br) (accessed on 7 April 2021). For all databases, the search filters were based on three complementary levels: (i) Ozone, (ii) Liver, and (iii) Antioxidant, which were combined using Boolean connectors [AND]. Search filters were initially developed for PubMed. The search algorithms [MeSH Terms] and [TIAB] were applied to identify the indexed records and those recently published in an indexing process, respectively. In addition, a back search (manual search) was performed, in which the reference list of each included study was manually screened for additional eligible studies that were not retrieved by our search. The descriptors created as a search strategy were detailed in the Supplementary Materials (Table S2).

### 2.3. Eligibility Criteria

After record identification in the three databases, the duplicate studies were removed. Then, an initial selection based on the title and abstract was performed. In this initial selection, we included pre-clinical studies in murine models that assessed the effects of ozone exposure on the oxidative balance in hepatic damage. All studies that evaluated the oxidative stress and antioxidant potential of ozone in liver cells from murine models were included in this research. All timings, frequencies, and dosages of ozone exposure were eligible for inclusion. Secondary (literature reviews, letters to the editor, case studies, comments, and editorials) and in vitro studies were also excluded. After the initial screening, all relevant studies were recovered in full text and evaluated using the eligibility criteria. We excluded studies that either had no full text available or did not meet the criteria described above.

### 2.4. Data Extraction and Management

The kappa test was performed for the selection (kappa = 0.925). Publication data were extracted through standardization information such as (1) publication characteristics and animal models (author, country, ethics committee, statistical analysis, lineage, sex, age, and weight); (2) cell oxidative stress (oxidative markers; antioxidant enzymes: superoxide dismutase (SOD), catalase (CAT), glutathione S-transferase (GST); ROS and free radicals; (3) inflammatory markers, inflammatory cells, and liver injury parameters. Then, data were compared between reviewers, and conflicting information was corrected. The features collected from the studies and used for their evaluation are presented in Tables S3–S5.

### 2.5. Bias Analysis

The quality of the studies was assessed through the risk of bias (RoB), a tool from the Systematic Review Centre for Laboratory Animal Experimentation) (SYRCLE), designed specifically for animal studies [12]. The following methodological domains based on RoB were evaluated considering the following: Q1 and Q2 consider selection bias; Q3 considers performance bias due to knowledge; Q4 considers detection bias due to knowledge of interventions by outcome evaluators; Q5 considers attrition bias (quantity, nature, or processing of incomplete results data); Q6 considers reporting bias due to selective result reporting. In addition, we asked eight additional questions that contributed to the judgment of the studies; Q7 considers that the conditions in which the animals were kept were reported (temperature, humidity, light/dark cycles, water, and food); Q8 considers whether information about the intervention is complete (dose, time and interval of exposure of the intervention); Q9 considers allocation information (individual, collective, how many per allocation); Q10 considers whether the study was approved by the ethics committee; Q11 considers whether the study reports dropouts and/or exclusions from any group and the reason; Q12 considers whether the methodology used to obtain the results is validated if it is available, or if it is replicable; Q13 considers whether the statistical methods used were reported; Q14 considers whether the study directly addresses the review issue. The items in the RoB tool were scored with “yes” (low risk of bias); “no” (high risk of bias); or “unclear” (indicating that the item was not adequately reported and, therefore, the risk of bias was unknown). Based on these items, we constructed a figure using the Review Manager 5.4 program, centered on Cochrane Collaboration (RoB 2.0), to demonstrate the risk of bias across all studies included.

## 3. Results

### 3.1. Selection of PRISMA-Guided Studies

Our search strategy allowed us to retrieve 931 studies (PubMed: 221; Scopus: 378; Web of Science: 332). After removing 290 duplicates, 624 studies were excluded due to inappropriate topics selected by reading the titles and abstracts. A total of 470 studies were read in full (full text), and 456 were excluded using the eligibility criteria. After reading the bibliographic references of the 14 selected articles, 5 studies were added, totaling 19 studies (Figure 1).

### 3.2. Animal Model Characteristics

**Figure 2:**
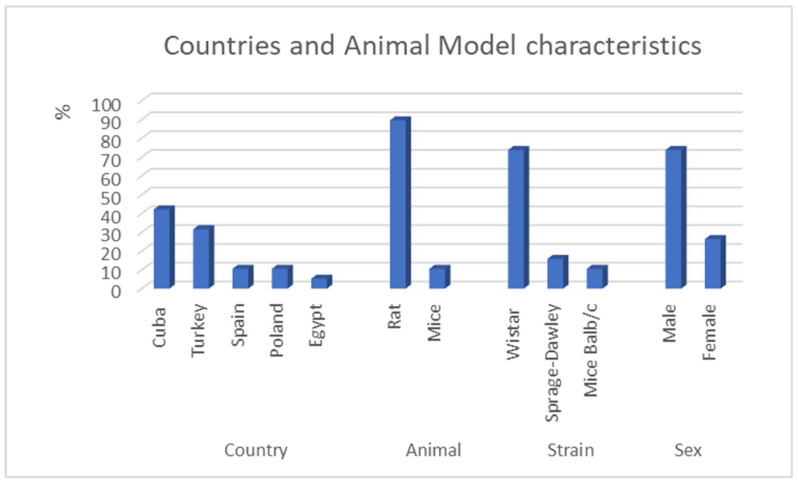
Countries and animal model characteristics of the studies included in this review.

The general characteristics of the selected studies and experimental models are shown in Table S3. The studies were published between 1996 and 2020 and were carried out in several countries, mainly Cuba, followed by Turkey, Spain, Poland, and Egypt (Figure 2). Rats were the main animal model used in the studies (*n* = 17; 89.47%), followed by mice (*n* = 2; 10.53%). Among strains of rats, most studies used Wistar (*n* = 14; 73.68%) and Sprague-Dawley rats (*n* = 3; 15.79%) (Figure 2). Among mice, there was Balb/c (*n* = 2; 10.53%). The experimental animals analyzed were male (*n* = 14) and female (*n* = 5) (Figure 2). All rat studies reported the weight and age of the animals, which ranged from 172 to 300 g, aged 3 to 6 months, respectively (Table S3). In mice, the weight of the animals ranged from 18 to 20 g, and these studies did not report age (Table S3).

### 3.3. Methods Used to Cause Liver Injury

Different methods were used to promote liver injury. In most studies, the ischemia/reperfusion technique was used, corresponding to 26.32% (*n* = 5), followed by LPS (lipopolysaccharide), 15.79% (*n* = 3); carbon tetrachloride (CCl_4_), 10.53% (*n* = 2); and cadmium (Cd). In addition, fecal material, acetaminophen, methotrexate, alcohol, iron dextran, aging, and ionizing radiation corresponded to 5.26% (*n* = 1, each) and mandibular defect. Mandibular bone defect increases oxidative stress as bone damage increases free radical production, contributing to oxidative damage in the liver [13].

### 3.4. Ozone Exposure Characteristics

The doses of ozone applied ranged from 0.2 mg/kg to 1.2 mg/kg. Ozone concentrations ranged from 3.8 to 67 µg/mL (Table S4). Ozone administration routes were performed intraperitoneal, corresponding to 57.89% (*n* = 11) and 42.11% rectal (*n* = 8). In 89% (*n* = 17) of the studies, the application of ozone occurred daily during the period of the experiment. The duration of ozone treatment ranged from 1 to 450 days, with most of the studies using between 4 and 6 days. The duration of treatments can be divided into three-time intervals: 1 to 10 days (*n* = 10; 48%), 15 to 27 days (*n* = 8; 38%), and 450 days (*n* = 3; 14%) (Table S5).

### 3.5. Outcomes

#### 3.5.1. Ozone Exposure and Metabolism Redox

Among oxidative markers, all studies that analyzed malondialdehyde (MDA) after liver injury showed there was a reduction in MDA levels (52.6% *n* = 10), revealing that ozone treatment prevented oxidative damage in liver tissue, strengthening the antioxidant defense system. There was a reduction in the 4-hydroxyalkenals (4-HDA) (15.78% *n* = 3), hydrogen peroxide (H_2_O_2_) (10.53%; *n* = 2), diene conjugate (CD) (5.26%; *n* = 1), and protein carbonyls (PC) (5.26%; *n* = 1) (Table 1).

Our results showed that about 70% of the doses applied were between 0.5 and 1 mg/kg. In our review, some studies analyzed the pro-oxidant and antioxidant enzyme capacity. It was observed that the enzyme-like myeloperoxidase (MPO) (5.26% *n* = 1), NADPH oxidase (NOX) (10.5% *n* = 2), and xanthine oxidase (XOD) (10.5% *n* = 2) were all reduced after ozone exposure.

Some studies (68% *n* = 13) also showed that there was an increase in the total antioxidant enzymes after ozone exposure. One antioxidant enzyme evaluated in this study was SOD (57.89% *n* = 11). SOD isoforms were also analyzed regarding Cu-Zn-SOD (5.26% *n* = 1) and Mn-SOD (5.26% *n* = 1). Other antioxidant enzymes evaluated were CAT (47.37% *n* = 9), GST 15.79% *n* = 3), glutathione (GSH) (36.84% *n* = 7), oxidized glutathione (GSSG) (10.53% *n* = 2), and glutathione peroxidase (GPx) (31.57% *n* = 6). In nine studies, there was an increase in SOD (82%), and in two studies, there was a reduction in SOD after ozone exposure. In the group treated with ozone exposure, there was an increase in endogenous SODs returning to the normal state. This implies cellular protection by reducing the availability of superoxide anion and reducing liver damage. In cases where liver damage increased SOD, ozone preconditioning reverted SOD levels to normal levels, indicating that ozone establishes redox balance.

In three studies (15.8%), there was no effect of ozone on the CAT content. In two studies (10.5%), there was an increase, and in four studies (21%), there was a reduction in the CAT. Concerning glutathiones, in two studies (10.5%), GST increased, and in one study, (5.26%) there was no effect of ozone on GST content. In seven studies, there was an increase in GSH (36.8%); in one study (5.26%), the GPX was not changed; in one study (5.26%), the GPX decreased; and in four studies, the GPX increased (21%). In two studies (10.5%), the GSSG was reduced. Due to ozone treatment, it is observed there is an adaptation of the tissues to oxidative stress by inducing enzymes or activating the metabolic pathways. Thus, maintaining a redox balance with an increase in glutathione levels and a decrease in lipid peroxidation regulates the cell’s thiol-redox status.

#### 3.5.2. Ozone Exposure and Inflammation

Our results showed that the most frequent inflammatory parameters reported were cellular markers, cytokines, morphological changes, and biochemical markers. Our review revealed that ozone exposure was efficient in controlling the inflammatory process by the decrease in the total leukocyte number, especially macrophages (Kupfer cells) and neutrophils (21% *n* = 4). Consequently, important pro-inflammatory markers produced by these cells also presented a decreased expression, like tumor necrosis factor-α (TNF-α) (15.8% *n* = 3) and interleukin 1beta (IL-1β) (5.26% *n* = 1).

**Figure 3:**
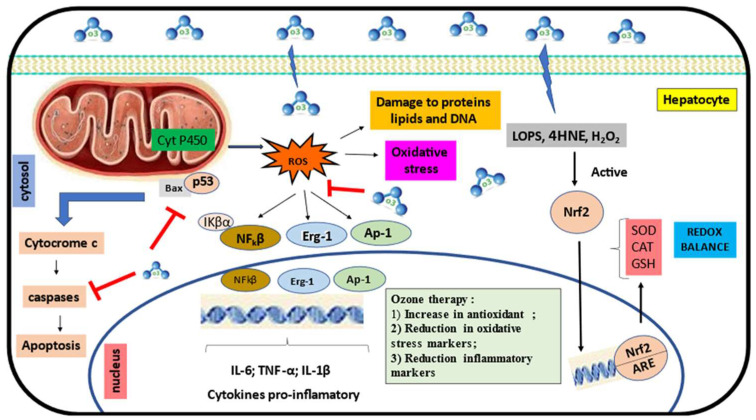
The effect of ozone on the protection of liver cell damage in a murine model.

Our results showed that the main morphological changes present in the tissue associated with the inflammatory process were periportal inflammation (15.8% *n* = 3), vascular congestion (10.5% *n* = 2), and cell death like necrosis (10.5% *n* = 2), with ozone exposure being efficient to reduce this damage, possibly due to its anti-inflammatory and antioxidant capacity (Figure 3).

In addition, chemotactic markers in inflammatory cells were also identified. Neopterin is an immunity-associated biochemical in cells produced in monocytes/macrophages that allows for monitoring the progression of inflammatory markers [31,32]. In our study, ozone treatment reduced macrophages in liver tissue, decreasing neopterin levels and consequently reducing inflammation. In addition, Phospholipase A was reduced (5.26% *n* = 1) after ozone exposure, thus reducing the hydrolysis of phospholipids and, consequently, the processes associated with inflammation.

#### 3.5.3. Secondary Outcomes

Regarding secondary outcomes, we observed the labeling of different enzymes, intracellular activators, and polysaccharides. In this context, ozone exposure promoted an increase in activity of calcium-dependent ATPase (Ca^2+^ATPase) (5.26% *n* = 1) and reduced Ca^2+^ levels (5.26% *n* = 1), markers that are altered during liver damage. Another enzyme that was reduced by ozone exposure was Calpain, decreasing oxidative stress and damage inside the cells (5.26% *n* = 1). In addition, ozone treatment increased adenosine triphosphate (ATP) and adenosine diphosphate (ADP) (5.26% *n* = 1), providing more energy to cells to maintain high metabolism during the inflammatory process. Some markers that show an overload in liver tissue were also described in our review, with emphasis on aspartate aminotransferase (AST) and alanine aminotransferase (ALT); these markers were reduced after ozone exposure.

In addition, ozone exposure proved to be effective in maintaining hepatic glycogen content, indicating that ozone offers protection against glycogen reduction (5.26% *n* = 1%), preventing its degradation into lactate, thus decreasing intracellular acidosis associated with anaerobic glycolysis. In addition, our results showed that there was a reduction in lipofuscin after ozone exposure. Most of the studies included in this review attributed these results to the antioxidant capacity of ozone.

The therapeutic action of ozone occurs through the formation of ROS and lipid oxidation products (LOPs), and both act in different phases like a molecular beacon. While ROS acts immediately and is neutralized by antioxidant systems, LOPs are distributed throughout the tissues and have the function of reducing potential toxicities. The production of LOPs occurs after the oxidation of polyunsaturated fatty acids (PUFAs) in the cell membrane. ROS are produced in mitochondria through cytochrome P450. Both can be damaged in the cells, but when they are released in lower or moderate doses, they can activate Nrf2, which regulates gene expression through the antioxidant response element (ARE). The protein (keap1)/Nrf2 ARE signaling pathway primarily regulates anti-inflammatory gene expression and inhibits the progression of inflammation. Under moderate oxidative stress induced by ozone, Nrf2 is translocated to the nucleus, where it binds to ARE genes. This leads to the inhibition of the NF-κB pathways, reducing the expression of pro-inflammatory cytokines. Therefore, ozone decreases the levels of the pro-inflammatory markers IL-6, TNF-α, and IL-1β.

#### 3.5.4. Risk of Bias and Methodological Quality Assessments

**Figure 4:**
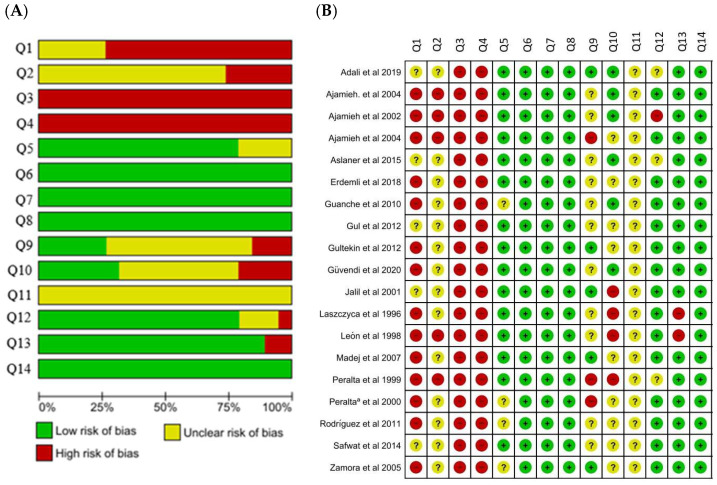
(**A**) Results for the risk of bias and methodological quality indicators for all studies included in this systematic review that evaluated the effect of ozone exposure on oxidative stress in liver tissue. The items in the Systematic Review Center for Laboratory Animal Experimentation (SYRCLE) Risk of Bias assessment were scored with “yes”, indicating low risk of bias; “no”, indicating high risk of bias; or “unclear”, indicating that the item was not reported, resulting in an unknown risk of bias. Q1 and Q2 consider selection bias; Q3 considers performance bias due to knowledge; Q4 considers detection bias due to knowledge of interventions by outcome evaluators; Q5 considers attrition bias (quantity, nature, or processing of incomplete results data); Q6 considers reporting bias due to selective result reporting. In addition, we added seven additional questions that contributed to the judgment of the studies; Q7 considers that the conditions in which the animals were kept were reported (temperature, humidity, light/dark cycles, water, and food); Q8 considers whether information about the intervention is complete (dose, time and interval of exposure of the intervention); Q9 considers allocation information (individual, collective, how many per allocation); Q10 considers whether the study was approved by the ethics committee; Q11 considers whether the study reports dropouts and/or exclusions from any group and the reason; Q12 considers whether the methodology used to obtain the results is validated, available, or replicable; Q13 considers whether the statistical methods used were reported; Q14 considers whether the study directly addresses the review issue. (**B**) Risk of bias summary-review authors’ judgments about the risk of bias items for each included study. Green: low risk of bias. Yellow: unclear risk of bias. Red: high risk of bias. Refs. [5,13,14,15,16,17,18,19,20,21,22,23,24,25,26,27,28,29,30].

Detailed results for the bias analysis are shown in Figure 4. No study met all the methodological criteria analyzed. Regarding selection bias, the sequence generation process presented a high risk of bias in 73.68% of the studies (*n* = 14). In terms of allocation concealment, 26.32% (*n* = 5) presented a high risk of bias, while 73.68% (*n* = 14) presented an unclear risk. None of the articles reported random accommodation or blinding of caregivers (binding of participants and personnel, blinding of outcome assessment, respectively), and the outcome was assessed as presenting a high risk of bias. Incomplete outcome data were adequately addressed in 78.95% of the studies (*n* = 15); all studies were free from selective reporting (*n* = 19) and clear data on the conditions of the animals (*n* = 19). Regarding intervention, 100% of the studies (*n* = 19) presented clear data. In terms of the unit of allocation, 57.89% of the studies (*n* = 11) presented unclear data, 26.32% of the studies (*n* = 5) presented a low risk of bias, and 15.79% of the studies (*n* = 3) presented a high risk of bias. Regarding ethical approval, 47.37% of the studies (*n* = 9) presented unclear data, 31.58% of the studies (*n* = 6) presented a low risk of bias, and 21.05% (*n* = 4) presented a high risk of bias. All withdrawal and exclusion studies presented a low risk of bias. In addition, three studies (15.79%) presented unclear data, 78.95% (*n* = 15) presented a low risk of bias, and 5.26% (*n* = 1) had a high risk of bias for tool validation. In 89.47% of the studies (*n* = 17), information was not clear concerning statistical methods, while 10.53% (*n* = 2) had a high risk of bias. In the applicability item, 100% of the low risk of bias was obtained. In 100% of the studies (*n* = 19), they presented an unclear other bias.

## 4. Discussion

Ozone is formed by the molecular disruption of oxygen (O_2_) in the stratosphere, by ultraviolet radiation from the sun, and natural electrical discharges [8,9]. It is considered an unstable pollutant in nature and is toxic, which contributes to several respiratory diseases and damage to the skin, among others [8]. Ozone is an oxidant that acts on macromolecules and intracellular oxidation pathways. Exposure to ozone induces oxidative stress, resulting in intracellular and extracellular changes in ROS levels, where increased ROS levels lead to tissue damage and affect mitochondrial structure and function, potentially causing cell damage [33]. Ozone also activates the interaction of cellular signaling networks, including membrane receptors, intracellular kinases and phosphatases, and transcription factors regulating inflammatory genes, inducing injuries and inflammation through the activation of NF-κB [33]. Thus, ozone triggers the generation of ROS, activates mitogen-activated protein kinases (MAPK), epidermal growth factor receptor (EGFR), Nrf2, and modifies the associated signaling pathways and transcription factors [34]. However, there is a distinction between airborne pollutant ozone (O_3_) and “medical gaseous ozone”, which is generally administered in a balanced O_2_/O_3_ mixture [35]. This has broad benefits when used at appropriate concentrations and time, as the application of ozone in a controlled manner is capable of stimulating the production of antioxidant and anti-inflammatory agents, preserving cellular redox balance, mitochondrial function and the regulation of transcription factors [7].

The knowledge about redox metabolism and the role of oxidative stress in liver diseases indicates that there is a direct relationship between redox imbalance and the inflammatory process. It is known that the inflammatory process and oxidative events are potentially mediated by pro-inflammatory cytokines such as TNF-α, interferon-gamma (IFN-γ), IL-1 and interleukin-6 (IL-6). Cytokines can stimulate intense reactive species production in liver tissue. Currently, the search for alternative therapies that reduce liver damage has grown increasingly, indicating a promising market [5]. Ozone exposure has become a promising therapy as it acts by stimulating the immune system locally or systemically, leading to a fast and safe tissue repair process [5]. However, there is a knowledge gap regarding the understanding of the entire liver recovery process, especially concerning the effect of these alternative therapies on oxidative stress and the inflammatory process in liver diseases. Therefore, in our study, we performed a systematic review to investigate the use of ozone exposure to treat hepatic damage, with a focus on the redox metabolism and inflammatory process in murine models.

### 4.1. Characteristics of the Study and the Animal Model

Despite being investigated throughout the decades [36], only in a few countries is ozone regulated as a therapy in medical practice, such as in Italy, France, Greece, Turkey, Cuba, Russia, China, Portugal, Japan, Spain, and the United States of America [37]. This fact may justify our findings by the predominance of studies in countries such as Cuba, followed by Turkey and Spain. This is possibly because it was in these countries that the regulation for the use of ozone exposure occurred earlier than other countries. In Cuba, there are 39 clinical centers for ozone exposure regularly attending to the population within its largest hospitals, incorporating ozone exposure into their care routines since 2009 [38]. Between 2000 and 2020, there was an increase in studies because of the regularization of ozone exposure in the health system [38]. Another interesting element identified in our review was the majority use of male rats as experimental models, probably because males present fewer hormonal fluctuations and therefore fewer behavioral changes than females [39]. One of the main advantages of using a murine model for liver injury is the ability to obtain samples to perform oxidative stress analyses and inflammation marker quantification, and it is even easier for histopathological and biochemical follow-up of liver injury [40]. All of these points are crucial to understand the main cellular mechanisms that are activated inside the cells after exposure to ozone. In addition, they are affordable, widely available, and easy to maintain and handle, and a variety of animals can be used for experiments generating a greater degree of reliability in the results [41]. However, the most used animal was the Wistar rat, weighing between 180 and 300 g, possibly because this animal has greater weight and is easier to manipulate, and more tissue may be obtained for analysis. Thus, for the induction of liver damage in murine models, the main methodology used was the induction of ischemia. The second most used method was the induction of inflammation using LPS. Liposaccharides are endotoxins of low acquisition cost and are highly effective in inducing inflammation, which makes the LPS model a viable alternative in studies that require modulation of the immune system [42].

### 4.2. Intervention Characteristics

Most studies presented intraperitoneal administration of ozone, which is a simple application technique with reduced gas loss [23]. Usually when different drugs are tested in the preclinical study, the intraperitoneal route is the most used, mainly due to the difficulty of approaching animal veins [38]. Another route used in our studies is rectal insufflation. Rectal insufflation of ozone is easy, painless, and less invasive. Several studies have demonstrated the effectiveness of this application, thus being the most common route in different types of treatments [43,44]. Rectal ozone is a safe, effective, low-cost, and simple option, making the results found in the studies that used this route easier to translate for the human context because this practice has been adopted in clinical trials around the world [45].

Another important feature of the reported intervention was the dose. The most used ozone dose was 1 mg/kg (concentration of 50 μg/mL), because it represents a concentration of 50 μg/mL, and a concentration greater than 60 or 80 has a toxic effect [38]; so, it used the highest dose of ozone that does not cause intoxication in the animal. Our findings showed that a dose lower than 0.14 mg/kg (concentration of 40 μg/mL) did not affect treatments, demonstrating that the hepatic effect is dose dependent. Concentrations ranging from 10 μg/mL to 50 μg/mL are safe and effective [38]. Thus, at low concentrations and doses, ozone acts as a bioregulator of redox balance, improving antioxidant capacity and activating important anti-inflammatory pathways like NrF2 [46,47], protecting cells from oxidation and suppressing inflammatory responses.

Associated with information on doses, another important feature of the intervention is frequency. Most studies reported that ozone treatment was performed once a day, possibly because the frequency is related to the duration of the treatment, and the duration of the experiments were very short, highlighting between 5, 10, and 15 days. The number of treatment sessions and the dose of ozone administered depends on several factors, such as the patient’s general condition, age, and disease [38]. However, a therapeutic protocol adapted to each patient is necessary as it depends on the clinical evaluation and whether the protocol is validated and recognized by the international scientific community of ozone exposure [48].

### 4.3. Ozone Exposure and Redox Metabolism and Inflammation Process

Excessive generation of free radicals promotes an imbalance between oxidant and antioxidant products inside the cells, promoting the oxidative stress process [49]. Generally, during liver diseases, the generation of oxidative stress in the tissue is associated with the decoupling of the electron transport chain [50]. This process leads to the oxidation of biomolecules with consequent loss of their biological functions and/or homeostatic imbalance [49]. Thus, identifying alternative therapies involved in the control of redox metabolism in liver diseases may represent a rational and useful strategy for developing an antioxidant therapy to treat hepatic lesions. In this sense, ozone exposure is currently the most promising non-invasive therapy to recover hepatic tissue. In this review, we observed that the methodological analyses carried out focused on lipidic and protein oxidative markers and pro- and antioxidant enzymes. It is important to highlight that free radicals and ROS are very unstable, the quantification of oxidative stress in the tissue is challenging, and it is very common to quantify the peroxidation and protein oxidation to quantify the level of damaged tissue.

In our review, there was a predominance of analyses of the lipidic peroxidation and markers like hydroperoxides, MDA, and 4-HDA. In all of the studies, there was a decrease in the production of these markers after ozone exposure. This shows that the ozone is efficient in controlling lipoperoxidation and, subsequently, the attack of free radicals and ROS in cellular membranes. A positive effect of ozone exposure on redox metabolism may occur due to ozone acting as a pro-oxidant modulator by inducing secondary messengers that are aldehydes and hydroxy hydroperoxides (ozone peroxide), thus forming H_2_O_2_ and a second aldehyde-4-hydroxynonenal (4-HNE), which develops an adaptive and regulated response in the antioxidant systems, controlling oxidative stress via an increase in the expression of the antioxidant enzymes. In addition, there is a direct relationship between the oxidative markers and the inflammation process; ozone exposure achieves modulation of the Nrf2 and NF-kB pathways [51]. These pathways, especially NrF2, are involved in the initiation of mild oxidative stress, capable of eliciting cell antioxidant expression without causing stress-related injury. Therefore, it is very important to establish a good treatment protocol for ozone exposure to ensure the production of controlled pro-oxidant molecules without promoting cellular damage. In addition, the administration of doses between 10 μg/NmL and 50 μg/NmL stimulates antioxidant enzymes including superoxide dismutase, glutathione peroxidase, and glutathione transferases, strengthening the antioxidant enzymes system [8,35].

Another important oxidative marker described in the studies included in this review was protein carbonyl. This marker is related to the oxidation of the proteins that promote a modification of native amino acid side chains in carbonyl proteins, which can lead to a loss of protein function [52]. These promote the misfolding of proteins and compromise their functions, leading to their inactivation [53]. Ozone reduces the number of carbonyl proteins within cells through upregulation of heat shock protein 90 (HSP90), which prevents the insertion of carbonyl groups into the primary structure of proteins and, consequently, prevents their misfolding [28]. Oxidative stress inside the cells is an important factor in promoting unwanted protein misfolding. When this protein accumulates inside the cells and organelles, it can produce various disorders [9]. On the other hand, ozone exposure reduced the deleterious biochemical and histopathological effects through increased total antioxidant and capacity and decreased oxidative markers, decreasing the formation of bad misfolding proteins. In addition, [7] showed that ozone treatment induces moderate oxidative stress by activating Nrf2 in the nucleus, where it binds to the ARE elements of genes encoding important antioxidant enzymes. This modulates protein degradation systems, showing that ozone exposure has a high potential to coordinate the production and elimination of the negative misfolding proteins inside the cells.

Our findings showed the use of ozone exposure can reduce pro-oxidant enzymes such as MPO, NOX, and XOD. These enzymatic pathways are directly linked with the increase in oxidative markers like hydrogen peroxide and ROS, which are responsible for attacking cellular membranes, proteins, and DNA. This can be explained by the fact that after treatment with ozone, there is an increase in the superoxide dismutase and catalase activity levels. These enzymes are responsible for accelerating the passage of the electron and, consequently, promoting a reduction in the time and quantity of the superoxide ion, H_2_O_2_, and ROS [54]. These are harmful molecules produced during electron transportation in inner mitochondrial membranes [55]. In this sense, oxidases like NOX and XOD play an important role in redox signaling [56,57], especially by increasing the production of ROS, anion superoxide, and hydrogen peroxide by the mitochondrial NOX activity and XOD activation, respectively. On the other hand, ozone exposure reduces the NOX activation and reduces xanthine accumulation and, consequently, reduces free radicals and ROS accumulation, which confirms its antioxidant effect.

Exposure to ozone can either positively or negatively impact cellular antioxidant levels. In studies where hepatic damage reduced the levels of antioxidant markers, ozone preconditioning increased cellular antioxidants (Table 1). Ozone preconditioning in some studies reduced SOD, CAT, and GPx levels to normal levels when hepatic injury had caused an elevation in these markers, indicating that ozone establishes redox balance, minimizing tissue damage (Table 1). However, in other studies, ozone exposure had no effect on CAT, GST, and GPx levels due to the low ozone dose, thus not inducing moderate oxidative stress and not stimulating the production of antioxidant enzymes [14]. Nevertheless, when hepatic injury elevated antioxidant marker levels, ozone preconditioning reduced these levels close to control values, restoring redox balance (Table 1).

The action of antioxidant enzymes such as SOD, CAT, GPx, glutathione reductase (GR), and GST activated by ozone through the positive regulation of Nrf2 neutralizes pro-oxidants linked to inflammation such as xanthine oxidase [58]. These results showed us that ozone exposure has an important antioxidant function by stimulating oxidative preconditioning or improving adaptation to oxidative stress and increases the activity of antioxidant enzymes to address ROS-mediated pathophysiological conditions [59]. The reaction of ozone with the cell membrane lipids produces H_2_O_2_ that can function as mixing stimulants, demonstrating the immunomodulatory effect of ozone through the status of antioxidant lipid generators [35]. LOP dissociates Keap1-Nrf2 and activates the nuclear factor erythroid 2-related factor 2- antioxidant response element (Nrf2-ARE) pathways, thereby increasing antioxidant enzymes after ozone exposure. Their activation indicated that the oxidative and inflammatory processes are deeply related during the damaged hepatic cells and that ozone exposure can be a good therapy to control the oxidative process inside the cells.

The Nrf2-ARE pathway described above is also involved in the modulation of the inflammatory process by modulating macrophage phagocytic activity and blocking the NF-κB pathways, reducing the release of microbial products and cytokines. This includes IL-1, IL-6, and tumor necrosis factor, showing that there is a relationship between inflammation and oxidative stress [60]. In addition, the pro-inflammatory response is associated with the formation of ROS and the consequent generation of oxidative stress [61]. Therefore, in general, ozone exposure is an interesting alternative for the treatment of liver diseases with a focus on oxidative balance mechanisms. In addition, ozone exposure proved to be effective in reducing cell degeneration and necrosis. Ozone blocks apoptotic processes by reducing the expression of caspases, TNF-α, Bcl-2-associated protein X (Bax), and p53 genes. These results are directly related to the decrease in the number of inflammatory cells, like neutrophils and Kupfer cells after ozone exposure. We already know that phagocytes hold an important role in the inflammatory and oxidative processes, and these cells represent a link between both processes involved in the development of different diseases.

### 4.4. Ozone Exposure and Other Markers

Ozone exposure increases ATP as it stimulates the Krebs cycle in the mitochondria, increasing oxidative carboxylation of pyruvate and stimulating the production of ATP [7]. In addition, ozone exposure increased Ca^2+^ ATPase activity and reduced calcium and calpain levels. Our results showed that ozone exposure promoted a reduction in calcium content. Calcium is a messenger associated with hepatic processes and its dysregulation is related to liver injury [62], so the control of intracellular calcium is considered a therapeutic target for liver injury. Ca^2+^ ATPase is a transport protein present in the plasma membrane that transports Ca^2+^ ions out of the cytoplasm vital for regulating the amount of Ca^2+^ within cells [63]. The increase in Ca^2+^ ATPase activity through ozone treatment will reduce the calcium content inside the cell. In addition, ozone promoted the reduction of another protease called calpain. Calpains are calcium-dependent cysteine proteases [64], and the involvement of calpain in liver dysfunction depends on its mediation of oxidative stress and inflammation, which are the most important contributors to the initiation and progression of liver dysfunction [65]. Calpain plays an essential role in liver disease, and its inhibition may protect against liver damage [66]. Therefore, ozone exerted a beneficial effect in reducing calpain.

Elevated liver enzymes AST and ALT occur due to damage to hepatocytes [67]. Studies point to the effectiveness of alcohol in induced damage [68] using drugs such as paracetamol [69], demonstrating that AST and ALT levels increased regardless of the cause of the injury [70]. In our studies, ozone exposure reduced ALT and AST levels in ischemia/reperfusion-induced liver injury. As ALT and AST are produced by hepatocytes and only released in case of damage to these cells, we can highlight, in this context, the cytoprotective effect of ozone on hepatocytes reducing the release of ALT and AST. Liver injuries reduce glycogen levels through increased oxidative stress and inflammation [71]. The maintenance of glycogen is important for proper cellular functioning as its reduction impacts homeostasis, leading to oxidative stress [71]. Ozone preserves the glycogen content, thus reducing liver damage, generating the accumulation of cytochrome P450 system enzymes and antioxidant enzymes and increasing the number of glycogen molecules [9]. Furthermore, the oxidative preconditioning ability of ozone has been reported to preserve the liver glycogen content and reduce lactic acidosis [71].

### 4.5. Methodological Quality and Risk of Bias

The risk of bias analysis was performed individually to ensure the validity of the findings and to assess the methodological quality of the studies, demonstrating that the application of standardized protocols is essential for the reproducibility and synthesis of the results. Bias analysis showed that key features such as blinding of participants (caregivers and outcome assessor) were not reported or unclear in the studies. In addition, some registries provided incomplete result data and insufficient information, which affects the accuracy of the results. It is important to emphasize that all types of reviews have limitations, and these limitations are clear and more evident in systematic review studies due to the use of specific tools to evaluate the quality of the evidence. In our review, the biggest limitation was the heterogeneity of the studies, which makes the task of comparing them difficult. The lack of information regarding the age of the animals was also neglected by most studies, which may be a reporting bias as it compromises the quality of the report.

We also observed that individual studies only analyzed a few oxidative markers, markers of the antioxidant system, pro-inflammatory cytokines, inflammatory cells, and morphological parameters. In individual studies, each element of methodological bias may be associated with variability in the objectives of different studies. However, it is important to emphasize that all types of reviews have limitations, and these limitations are most evident in systematic review studies, which extract information from primary studies to understand the process in its entirety. Thus, it is worth mentioning that our findings are important for understanding the mechanism of action of ozone and its therapeutic treatments, describing important points of bias. We hope to contribute to future studies, avoiding those elements of bias that impair the quality of the evidence.

## 5. Conclusions

Ozone exposure has a beneficial effect for animal models with liver injury through the decrease in oxidative stress in tissue and inflammatory marker expression, thus decreasing pathological processes such as degeneration and necrosis. In addition, ozone exposure regulates the Nrf2/ARE antioxidant pathway and blocks the NF-κB inflammatory pathway. This increases the expression of antioxidant enzymes and reduces the levels of pro-inflammatory cytokines (TNF-α, IL-1β), controlling the oxidative stress and inflammatory process. However, it is worth mentioning that the therapeutic function of ozone is also associated with the generation of moderate oxidative stress promoted by the activation of secondary messengers, which stimulate the production of antioxidant enzymes. However, future studies are needed to understand the mechanisms of ozone action, standardization of doses and concentrations, and exposure time for different liver injuries. Therefore, we hope that this review will be used as a guide for improving future research on ozone exposure in liver disease.

## Figures and Tables

**Table 1:** Main results of the action of ozone exposure on oxidative stress in liver tissue.

	Oxidative Markers					Pro−Oxidant Enzymes			Antioxidant Markers								Infl. Cells		Infl. Mark		Morphologic Parameters				Other Analyzes											
	MDA	H_2_O_2_	PC	4−HDA	CD (Conjugado dienos)	NOX(NADPH OXIDASE)	XOD	MPO	SOD	Cu, Zn−SOD	Mn−SOD	CAT	GSH	GST	GSSG	GPX	Kupfer Cell	Neutrofilos	TNF−α	IL−1β	Necrosis	Degeneration	Congestion	periportal Inflammation	Ca−ATPase	Ca^2+^	Calpain	Phospholipase A	Lipofuscin	Lactate	ATP + ADP	Ácido Urico	Glicogênio	Neopttirna	AST	ALT
Laszczyca, et al., 1996 [14]												=		=		=																				
León, et al., 1998 [15]	−								+			=	+												+			−							−	
Peralta, et al., 1999 [16]		−							+				+					−			−									−					−	−
Peralta, et al., 2000 [17]		−					−																								+				−	−
Jalil et al., 2001 [18]	−								+			=																		−		−	+			
Ajamieh et al., 2002 [19]				−			−										−									−	−								−	−
Ajamieh et al., 2004 [20]				−		−			+			−	+		−																				−	−
Ajamieh et al., 2005 [21]				−					+	−	+	−	+		−							−													−	−
Zamora et al., 2005 [22]	−											−		+		+			−																	
Madej et al., 2007 [23]									−			−																								
Guanche et al., 2010 [24]	−				−				+			+				+																				
Rodríguez et al., 2011 [25]	−								+					+		+																				
Gul et al., 2012 [26]	−					−			+							+					−													−	−	−
Gultekin et al., 2012 [27]	−								+								−		−			−	−	−											−	−
Safwat et al., 2014 [28]	−		−										+			−													−							
Aslaner et al., 2015 [29]	−							−					+				−	−	−	−		−		−											−	−
Erdemli et al., 2019 [13]	−								−			+	+																							
Adali et al., 2019 [30]																						−	−	−											−	−
Guvendi et al., 2020 [5]																								+												

Obs: PC=protein carbonylate; Infla marks= inflammatory markers; Infla. cells = Inflammatory cells.
+Increased−Reduced=Not effect

## Data Availability

The data are contained within the article and Supplementary Materials.

## Supplementary Materials

The following supporting information can be downloaded at: https://www.mdpi.com/article/10.3390/antiox13020212/s1, Table S1: Complete search strategy with search filters and the number of studies retrieved from databases PubMed-Medline e Scopus; Table S2: Full search strategy in PubMed and Scopus, including search terms and filters; Table S3: Animal model Characteristics; Table S4: Intervention characteristics ozone therapy and liver injury; Table S5: Frequency and duration of days of ozone treatments.
