# Supplementary material for: Ozone Exposure Controls Oxidative Stress and the Inflammatory Process of Hepatocytes in Murine Models
Source: Antioxidants (Basel). 2024 Feb 8;13(2):212. doi: 10.3390/antiox13020212 (PMC10886373; doi:10.3390/antiox13020212)
Supplement: Supplementary file 1 [file antioxidants-13-00212-s001.zip › antioxidants-2775534-supplementary.pdf]

Supplementary materials

**Table S1.** Complete search strategy with search filters and the number of studies retrieved from databases **PubMed-Medline e Scopus**.

**Table S1- Research Question Components following the PICO Strategy**

| Abbreviations | Parameters (description) | Question Components |
|---|---|---|
| P | Population | Murine models |
| I | Intervention | Ozone therapy |
| C | Comparison | Animals that did not receive ozone |
| O | Outcomes | Oxidative stress in liver tissue |

**Table S2- Full search strategy in PubMed and Scopus, including search terms and filters.**

| Data base | Descriptors | Items Found | Time | Date |
|---|---|---|---|---|
| P u b M e d | #1 Ozone Filter<br>("ozone"[MeSH Terms] OR Ozone therapy [TIAB]) | 15,779 | 07/04/2021 | 17:23 |
| | #2 Therapeutics Filter<br>("therapeutics"[MeSH Terms] OR therapy[TIAB] OR "therapeutic use"[Subheading] OR "therapeutic uses"[MeSH Terms] OR therapeutic use[TIAB]) | 8,934,939 | 07/04/2021 | 17:23 |
| | #3 Antioxidant filter<br>("antioxidants"[MeSH Terms] OR "antioxidants"[Title/Abstract] OR "oxidative stresses"[Title/Abstract] OR "antioxidative stress"[Title/Abstract] OR "oxidative stress injury"[Title/Abstract]) | 172,003 | 07/04/2021 | 17:24 |
| | **Total:** #1 ABD #2 AND #3 | 221 | 07/04/2021 | 17:25 |
| **Data base** | | **Items Found** | **Time** | **Date** |
| S c o p u S | #1 Ozone Filter<br>(TITLE-ABS-KEY ("ozone") OR TITLE-ABS-KEY ("Ozone therapy")) | 112,509 | 07/04/2021 | 17:33 |
| | #2 Therapeutics Filter<br>(TITLE-ABS-KEY ("therapeutics") OR TITLE-ABS-KEY("therapy") OR TITLE-ABS-KEY("therapeutic use")) | 4,851,800 | 07/04/2021 | 17:33 |
| | #3 Antioxidant filter<br>(TITLE-ABS-KEY(""antioxidants") OR TITLE-ABS-KEY ("antioxidants") OR TITLE-ABS-KEY ("oxidative stresses") OR TITLE-ABS-KEY ("antioxidative stress") OR TITLE-ABS-KEY ("oxidative stress injury")) | 650,154 | 07/04/2021 | 17:34 |
| | **Total:** #1 AND #2 AND #3 | **379** | 07/04/2021 | 17:34 |
| **Data base** | | **Items Found** | **Time** | **Date** |
| W E B O F S C I E N C | #1 Ozone Filter<br>TS=ozone OR TS=Ozone therapy | 96.550 | 07/04/2021 | 17:33 |
| | #2 Therapeutics Filter<br>TS=therapeutics OR TS=therapy OR TS=therapeutic use OR TS=therapeutic uses OR TS=therapeutic use | 2.852.755 | 07/04/2021 | 17:33 |
| | #3 Antioxidant filter<br>TS=antioxidants OR TS=antioxidants OR TS=oxidative stresses OR TS=antioxidative stress OR TS=oxidative stress injury | 661.260 | 07/04/2021 | 17:33 |
| | **Total: #1 and #2 and #3** | **332** | 07/04/2021 | 17:33 |

**Table S3 - Animal model Characteristics**

| Animal Model: Rat | Country | Strain | Sex | Age (months) | Weith (g) | Total number |
|---|---|---|---|---|---|---|
| Laszcyca , et al.1996 | Poland | Wistar | M | ? | 266 -299 | ? |
| León, et al. 1998 | Cuba | Sprague -Dawley | F | ? | 220–250 | 60 |
| Peralta, et al. 1999 | Spain | Wistar | M | ? | 250 - 300 | 18 |
| Peralta[a], et al. 2000 | Spain | Wistar | M | ? | 250 -300 | 56 |
| Jalil, et al. 2001 | Cuba | Sprague-Dawley | F | ? | 200–250 | 40 |
| Ajamieh, et al. 2002 | Cuba | Wistar | M | ? | 250–300 | 32 |
| Ajamieh, et al. 2004 | Cuba | Wistar | M | ? | 250–275 | 50 |
| Ajamieh, et al. 2005 | Cuba | Wistar | M | ? | 250–275 | 60 |
| Madje, et al. 2007 | Poland | Wistar | M | 4 | 164 -180 | 60 |
| Guanche, et al. 2010 | Cuba | Wistar | M | ? | ? | 60 |
| Gul, et al. 2012 | Turkey | Sprague-Dawley | M | ? | 200–250 | 27 |
| Gultekin, et al. 2012 | Turkey | Wistar | F | ? | 200–230 | 30 |
| Safwat, et al. 2014 | Egypt | Wistar | M | 3 | 180- 220 | 60 |
| Aslaner, et al. 2015 | Turkey | Wistar | M | ? | 250-300 | 18 |
| Erdemli, et al. 2018 | Turkey | Wistar | M | 3 - 4 | 200-250 | 36 |
| Adali, et al. 2019 | Turkey | Wistar | F | 4 - 6 | 190-250 | 48 |
| Guvendi, et al. 2020 | Turkey | Wistar | F | 4-6 | 190-250 | 48 |
| **Animal Model: Mice** | **Country** | **Strain** | **Sex** | **Age** | **Weith** | **Total number** |
| Reference | | | | | | |
| Zamora, et al. 2005 | Cuba | Balb /c mice | M | ? | 18-20 | 35 |
| Rodriguez, et al. 2011 | Cuba | Balb/c mice | M | ? | 18–20 | 50 |

**Table S4- Intervention characteristics ozone therapy and liver injury**

| Reference | Control | Liver injury | Applied dose (mg/kg) | Ozone concentration (µg/mL) | Route | Frequency | Duration (Day) |
|---|---|---|---|---|---|---|---|
| Laszczyca et al., 1996 | Cadmium | Cadmium | 0.14 ** | 40 | Intraperitoneal | Once a day | 10 |
| León et al., 1998 | $CCl_4$ | $CCl_4$ | 1 | 50 | Intraperitoneal | Once a day | 15 |
| Peralta et al., 1999 | Ischemia-reperfusion | Ischemia-reperfusion | 1 | 50 | Rectal insufflation | Once a day | 10 |
| Peralta et al., 2000 | Untreated | hepatic ischemia | 1 | 50 | Rectal insufflation | Once a day | 10 |
| Jalil et al., 2001 | $CCl_4$ | $CCl_4$ | 1 | 50 | Rectal insufflation | Once a day | 15 |
| Ajamieh et al., 2002 | ischaemia/reperfusion | hepatic ischemia | 1 | 50 | Rectal insufflation | Once a day | 15 |
| Ajamieh et al., 2004 | ischaemia/reperfusion | ischaemia/reperfusion | 1 | 50 | Rectal insufflation | Once a day | 15 |
| Ajamieh et al., 2005 | ischemia/reperfusion | hepatic ischemia | 1 | 50 | Rectal insufflation | Once a day | 15 |
| Zamora et al., 2005 | LPS | LPS | 0.2; 0.4; 1.2 | 3,8; 7,6; 22,8 ** | Intraperitoneal | Once a day | 5 |
| Madej et al., 2007 | LPS | LPS | 0.15 | 54 | Intraperitoneal | Once a day | 10 |
| Guanche et al., 2010 | Sepsis | Sepsis (fecal material) | ? | 10, 30; 50 | Intraperitoneal | Once a day | 5 |
| Rodríguez et al., 2011 | LPS | LPS | 0.2; 0.4; 1.2 | 3,8; 7,6; 22,8 ** | Intraperitoneal | Once a day | 5 |
| Gul et al., 2012 | acetaminophen | acetaminophen | 0.7 | 60 | Intraperitoneal | Once time | 1 |
| Gultekin et al., 2012 | Saline solution | ionizing radiation | 0.7 | 60 *** | Intraperitoneal | Once a day | 5 |
| Safwat et al., 2014 | Untreated | ageing | 0.6 | 67 ** | Rectal insufflation | 1) twice weekly/90 days once per week/ 450 days 2) three times weekly / 120 days | 450 |
| Aslaner et al., 2015 | Methotrexate | Methotrexate | 0.45 ** | 25 | Intraperitoneal | every day | 15 |
| Erdemli et al., 2018 | Mandibular defect | mandibular defect | 0.6 | 17 | Rectal insufflation | Once a day | 15 |
| Adali et al., 2019 | Alcoholic | ethyl alcohol | 0.5 | ? | Intraperitoneal | every day | 7 |
| Guvendi et al., 2020 | Iron overload | iron dextran | 0.5 | ? | Intraperitoneal | Six days a week | 27 |

* Not informed in the text; ** Calculated (Considered average weight); *** 60 mg/mL (change to microgram would be 60000 µg); LPS lipopolysaccharide

**Table S5: Frequency and duration of days of ozone treatments**

| Days | Frequency | Percentage (%) |
|---|---|---|
| 1 | 1 | 5,26 |
| 5 | 4 | 21,05 |
| 7 | 1 | 5,26 |
| 10 | 4 | 21,05 |
| 15 | 6 | 31,58 |
| 20 | 1 | 5,26 |
| 27 | 1 | 5,26 |
| 450 | 1 | 5,26 |
